# Psychosocial Factors, Stress, and Well-Being: Associations with Common Dermatological Manifestations in a Large Polish Cross-Sectional Analysis

**DOI:** 10.3390/jcm14113943

**Published:** 2025-06-03

**Authors:** Anna Kubrak, Anna Zimny-Zając, Sebastian Makuch, Beata Jankowska-Polańska, Wojciech Tański, Jacek C. Szepietowski, Siddarth Agrawal

**Affiliations:** 1 Labplus R&D, Wyspa Słodowa 7, 50-266 Wroclaw, Poland; a.kubrak@labplus.pl; 2 Medonet, Ringier Axel Springer Poland, Domaniewska St. 49, 02-672 Warsaw, Poland; anna.zimny-zajac@medonet.pl; 3 Department of Clinical and Experimental Pathology, Wroclaw Medical University, 50-367 Wroclaw, Poland; s.makuch@umw.edu.pl; 4 Faculty of Medicine, Wroclaw University of Science and Technology, 50-386 Wroclaw, Poland; beata.jankowska-polanska@pwr.edu.pl (B.J.-P.); wojciech.tanski@pwr.edu.pl (W.T.); 5 Department of Dermato-Venereology, 4th Military Hospital, 50-981 Wroclaw, Poland; 6 Division of Dermatology, Venereology and Clinical Immunology, Faculty of Medicine, Wroclaw University of Science and Technology, 50-386 Wroclaw, Poland

**Keywords:** skin signs and symptoms, stress, well-being, sociodemographic factors

## Abstract

**Background/Objectives:** Cutaneous manifestations can signal underlying systemic inflammation, potentially exacerbated by chronic stress and diminished well-being. While links between psychosocial factors and skin health are recognized, comprehensive data across diverse populations remain limited. This study aimed to quantify associations between self-reported stress management capabilities, sociodemographic factors (gender, age, education, urbanization, professional status), lifestyle factors indicative of well-being, and the prevalence of six common dermatological manifestations (pruritus, burning sensations, redness, rash, desquamation, sunburn) within a large Polish cohort. **Methods:** This cross-sectional study analyzed data from 27,000 adult participants (22,043 women, 4887 men) collected during the National Healthy Skin Test (2023) via an online questionnaire. Participants reported the frequency of dermatological symptoms, stress management practices related to skin health, and relevant lifestyle factors (indicators of well-being). Logistic regression analyses identified significant predictors for each skin manifestation. **Results:** Effective stress coping ability was significantly associated with a lower prevalence of all six investigated dermatological manifestations (*p* < 0.001 for all). Significant gender differences emerged: women reported more frequent redness and burning sensations (*p* < 0.001), while men reported more frequent rash, sunburn, and desquamation (*p* < 0.001). Younger age (18–24 years) was associated with increased rash, desquamation, and redness compared to older adults (>65 years), who reported fewer burning sensations and less pruritus. Higher education and residence in large urban centers (≥500,000 inhabitants) were associated with increased reports of specific symptoms like sunburn and redness. **Conclusions:** This large-scale study demonstrates a significant association between psychosocial factors, particularly self-reported stress management, and the prevalence of six common, self-reported dermatological manifestations across various sociodemographic groups in Poland. The findings underscore the potential importance of considering a biopsychosocial approach in relation to these common skin symptoms. Further research is warranted, but these results suggest that for such common, self-reported skin issues, integrating stress reduction strategies and considering sociodemographic contexts and well-being may be valuable considerations for potentially enhancing personalized patient care and warrant further clinical investigation.

## 1. Introduction

Stress has become an integral aspect of human functioning in the 21st century. It is defined as a psychological and physiological response to novel situations, challenging events, and external or internal demands placed upon individuals [1]. The prevalence of chronic stress has increased significantly in contemporary society, with extensive research documenting its detrimental effects on multiple physiological systems beyond psychological functioning, including cardiovascular, immune, and integumentary systems. Stress manifests with varying intensity and is influenced by multiple factors, including psychological (e.g., personality traits, past trauma, emotional regulation capacity), environmental (e.g., occupational stress, financial strain, social dynamics), biological (e.g., genetic predisposition, underlying health conditions), behavioral (e.g., substance use, levels of physical activity), and sociocultural (e.g., religious beliefs, experiences of discrimination and stigma) determinants. Stress can exert both adaptive and maladaptive effects on human functioning. It may enhance preparedness for action by facilitating cognitive and physiological arousal. However, stress, especially when chronic, may be associated with adverse health consequences, including heightened anxiety and an increased risk of developing stress-related disorders, such as hypertension [2].

Numerous studies have demonstrated a strong association between stress and an increased incidence of dermatological conditions, including psoriasis, acne, lichen planus, seborrheic dermatitis, rosacea, and atopic dermatitis. These conditions represent commonly encountered dermatological manifestations with established or suspected stress-related components, affecting substantial portions of the population and significantly impacting quality of life. For instance, Al’Abadie et al. [3] found that 70.2% of psoriasis patients reported a stressful event preceding disease onset, while 65.7% attributed subsequent exacerbations to stress. Furthermore, Shah et al. [4] revealed that 23.9% of adult acne patients had underlying chronic stress. Additionally, dermatological disorders themselves can act as significant sources of psychological stress, creating a bidirectional feedback loop that further deteriorates a patient’s overall health status. This heightened stress perception is likely linked to the chronic and visible nature of these skin diseases, which may potentially lead to social stigma and emotional distress [5].

Stress can also impair wound healing and increase the skin’s vulnerability to infections. For instance, individuals experiencing chronic stress, such as caregivers for Alzheimer’s patients, have been observed to have delayed wound healing compared to those with lower stress levels [6]. Furthermore, Rojas et al. demonstrated that stress impairs bacterial clearance during wound healing, resulting in a significant increase in the incidence of opportunistic infection. In this study, stressed mice exhibited delayed wound healing and a higher bacterial load compared to non-stressed controls [7]. These impairments in healing and barrier function may manifest clinically as various visible signs and symptoms, including redness, pruritus, and desquamation—alterations that not only affect physiological skin function but also impact patients’ psychological well-being and social interactions.

The underlying mechanisms linking chronic stress to skin disturbances involve the nervous, immune, and endocrine systems. For instance, chronic stress activates the hypothalamic-pituitary-adrenal (HPA) axis, leading to the release of cortisol and other stress hormones. Elevated cortisol levels can suppress immune function, impair skin barrier integrity, and disrupt homeostasis, thereby increasing susceptibility to various dermatological problems [8].

While the link between stress and specific dermatoses is established, less is known about how psychosocial factors relate to common, non-specific skin signs and symptoms across large, diverse populations. The primary goal of this study was therefore to analyze the relationship between psychosocial factors—particularly self-reported stress management capabilities and lifestyle indicators associated with well-being—and the prevalence of common, non-specific dermatological manifestations in a large Polish population cohort. These common skin signs and symptoms, while often not indicative of severe disease, can significantly impact quality of life, contribute to psychological burden, and represent a notable public health aspect due to their high prevalence and potential link to underlying psychosocial distress. We focused on six specific manifestations: pruritus, burning sensations, redness, rash, desquamation, and sunburn. These were selected because they are frequently encountered dermatological complaints in both clinical practice and population surveys, are often associated with physiological processes influenced by stress, are relatively easy for individuals to self-identify, and their occurrence can impact daily functioning and well-being, making them relevant indicators for exploring the psychodermatological axis in a broad population. Specifically, the study aimed to determine whether perceived stress coping abilities and well-being indicators were associated with the frequency of these skin manifestations and whether such associations differed across key sociodemographic groups. Based on the literature suggesting links between psychosocial well-being and skin health, and to address the identified knowledge gap for common symptoms in large populations, we hypothesized that: (1) effective self-reported stress management would be inversely associated with the prevalence of these common dermatological signs and symptoms; and (2) these associations would show significant variations based on sociodemographic factors. These findings could inform more personalized approaches to dermatological care that incorporate targeted psychosocial interventions based on patient profiles.

## 2. Materials and Methods

### 2.1. Data Collection

This cross-sectional study utilized data collected online from 27,000 adult participants as part of the ‘National Healthy Skin Test’ (*Narodowy Test Zdrowia Skóry*) survey conducted in Poland in 2023. The survey questionnaire, administered in Polish, was distributed via Medonet, a major social networking platform, to achieve broad reach across diverse demographic groups. The ‘National Healthy Skin Test’ questionnaire, from which the data for this study were drawn, was developed by a team of dermatologists, public health specialists, and survey methodologists from Medonet. Prior to its nationwide launch, the questionnaire underwent pilot testing with a sample of 100 adult internet users to assess clarity, comprehensibility, question flow, and technical functionality. Feedback from this pilot phase was used to refine the final version of the questionnaire used for data collection. The recruitment strategy involved targeted advertisements to reach a diverse Polish population across different age groups, geographic locations, and socioeconomic backgrounds. The study included adult (*≥18 years old*) Polish internet users, with no specific exclusion criteria beyond this basic requirement.

Participants were asked to provide demographic information, including age, gender, educational level, place of residence (degree of urbanization), and professional status. Additionally, they were requested to self-report the frequency of experiencing six specific skin signs and symptoms (pruritus, burning sensations, redness, rash, desquamation, sunburn) by selecting one of three options: ‘at least several times a month’, ‘occasionally—less than a few times a month’, or ‘never’. The six specific skin signs and symptoms investigated were selected based on several factors: they represent common dermatological complaints frequently encountered in clinical practice and population surveys; they are often associated with underlying physiological processes influenced by stress and environmental factors; and they significantly impact patient well-being and daily functioning. Furthermore, these manifestations are relatively easy for participants to self-identify and report in a questionnaire format.

Furthermore, respondents were asked to indicate the presence of specific features potentially determining skin appearance and quality, including: freckles, moles/birthmarks, discoloration/sun spots, visible blood vessels, dry skin, oily skin, fine wrinkles, crow’s feet, deep wrinkles, glabellar lines (“lion’s frown”), breakouts, high sensitivity to external factors/cosmetics, furrows, sagging facial contour, loose skin, erythema/redness, transverse forehead wrinkles, stretch marks, cellulite, and excessive body hair. In this section, participants could select one of the following responses: ‘yes’, ‘no’, or ‘I don’t know’. Stress management related to skin health was assessed using the specific question: ‘Do you try to reduce/deal with stress to take care of your skin?’ Participants selected one of three responses: ‘yes’, ‘sometimes’, or ‘no’.

Several lifestyle factors, considered indicators of well-being for this study, were also assessed via single-item questions with three response options each (typically ‘yes’, ‘sometimes’, ‘no’ or similar frequency scales). These included: adherence to a healthy, balanced diet; consumption of at least five servings of vegetables and fruit daily; adequate fluid intake relative to body weight; regular physical activity (at least 150 min/week); sleep duration (6–8 h/day); severely limiting alcohol consumption; non-smoking status; avoiding fast food and sweetened drinks; and use of dietary supplements. Specific questions regarding skin care practices, such as using appropriate cosmetics/care and undergoing treatments (e.g., massages, beauty treatments), were also included. These factors were selected based on established links between lifestyle behaviors and general health, which may indirectly influence skin condition. This comprehensive assessment allows for a detailed overview of self-reported skin characteristics, manifestations, and related lifestyle factors in Poland.

The survey was fully anonymous and voluntary, with no incentives provided. The estimated completion time ranged from 15 to 20 min. All participants provided informed consent online before participation, having been briefed on the study’s objectives. Data collected in this study were fully anonymized to ensure participant confidentiality and compliance with data protection regulations. The study received approval from the relevant Bioethics Committee. All procedures complied with the ethical standards of the Declaration of Helsinki. Data protection measures included complete anonymization and secure data storage.

### 2.2. Statistical Analysis

Frequency tables (n, %) were used for descriptive analysis. The chi-square independence test (χ^2^) compared frequencies between groups (e.g., gender differences for demographics and symptom frequency).

To identify predictors for the frequency of each skin manifestation, ordinal logistic regression was applied. A separate model was built for each of the six skin manifestations (pruritus, burning sensations, redness, rash, desquamation, sunburn), which served as the dependent variable (ordinal frequency: never, occasionally, frequently). The independent variables (predictors) considered in each model were: gender, age group, education level, degree of urbanization, professional status, the stress management question, and the well-being indicators and specific skin care factors (cosmetics use, treatment use) described above. Body mass (calculated from self-reported height and weight) was included as a potential confounder in all models.

The reference levels for categorical predictors in the regression models were set as follows: female gender, age 45–54 years, secondary education, urbanization level of a city with 50,000–99,000 inhabitants, and employment status of a pensioner. For the lifestyle/well-being indicators, stress management, and specific skin care factors (cosmetics, treatments), the response ‘sometimes’ was used as the reference category. These reference categories were generally selected based on having adequate subsample sizes within each category to ensure stable estimates. For variables such as stress management, lifestyle/well-being indicators, and specific skin care factors (where ‘sometimes’ was used as the reference), this choice also facilitates a more nuanced interpretation by allowing for comparisons of both affirmative (‘yes’) and negative (‘no’) responses against an intermediate or less consistent engagement in the behavior. This approach helps in understanding potential bidirectional associations relative to a common baseline. For sociodemographic variables like age or education, reference groups were chosen to represent common categories or those that allow for clinically or sociologically relevant comparisons (e.g., comparing other age groups to middle age or other education levels to secondary education). Regression coefficients (β) and odds ratios (OR) with 95% confidence intervals (CI) were calculated.

Sample size determination was conducted using standard statistical methods for population-based studies. Given that the true prevalence of these specific dermatological manifestations in the Polish population was unknown prior to this investigation, we adopted a conservative approach by assuming a population proportion of 50%, which yields the maximum sample size requirement. Calculations were performed with a 95% confidence level (corresponding to a Z-value of 1.96) and a desired precision of ±3% (margin of error = 0.03) for our prevalence estimates. Based on these calculations, the minimum representative sample size required for the study was determined to be *n* = 1068 individuals. This value is significantly smaller than our examined sample (*n* = 27,000). This substantial difference between the minimum required and actual sample size suggests that our study has strong statistical power and provides highly reliable results, with potential for even smaller margins of error than initially assumed.

The results were considered statistically significant when the type I error (α) did not exceed 5% (*p* ≤ 0.05). All analyses were performed using Statistica 13.5 (TIBCO Software Inc., Palo Alto, CA, USA) and Microsoft Excel 365 software packages.

## 3. Results

### 3.1. Demographic Characteristics

Our study population consisted of 4887 men (18.1%) and 22,043 women (81.7%); 70 respondents (0.3%) declared “other” gender. As previously reported [8], significant gender differences were observed across age groups, education levels, place of residence, and professional status. Briefly, women significantly outnumbered men in the 25–54-year age brackets, while men were more prevalent among those older than 55 years. Men more frequently reported primary or vocational education, whereas women more often reported secondary or higher education. Men were predominant in localities with 50,000–99,000 inhabitants and cities exceeding 500,000 residents, while women were more numerous in other urbanization categories. Men were most often retirees or pensioners, while women more frequently reported being employed, running their own businesses, being unemployed, or students (Table 1). These demographic differences provide important context for understanding the variation in skin manifestation prevalence described below.

### 3.2. Frequency of Skin Signs and Symptoms

We analyzed the frequency of six self-reported skin signs and symptoms—pruritus, burning sensations, redness, rash, desquamation, and sunburn—stratified by gender (Table 2). Our findings indicate significant differences in the prevalence of most of these manifestations between men and women. Pruritus frequency did not differ significantly between genders (*p* = 0.2098). Burning sensations and redness occurring at least several times a month were more frequently reported by women than men, as were occasional cases (*p* < 0.001 for both). In contrast, men significantly more often reported never experiencing burning sensations or skin redness. Rash, desquamation, and sunburn occurring several times monthly were significantly more common among men (*p* < 0.001 for all). Occasional rash and desquamation were more frequently reported by women. Understanding these gender-specific differences is crucial for developing targeted prevention and treatment strategies. To examine how these gender differences interact with other sociodemographic and psychosocial factors, we conducted multivariable analyses for each skin manifestation.

### 3.3. The Presence of Features Characterizing the Skin Condition

The analysis revealed significant gender-based differences in self-reported skin quality features. Women were more likely to report freckles, moles/birthmarks, discoloration/sun spots, visible blood vessels, dry skin, oily skin, and fine wrinkles than men. Additionally, deep wrinkles, glabellar lines (“lion’s wrinkle”), and skin breakouts were significantly more prevalent among women. They also exhibited higher sensitivity to external factors and cosmetics, along with a greater frequency of skin furrows, sagging facial contours, and loose (sagging) skin. Other conditions, including erythema (skin redness), transverse forehead wrinkles, stretch marks, cellulite, and excessive body hair, were also reported more frequently by women compared to men. Conversely, reporting taking steps to manage or reduce stress aimed at improving skin quality (‘yes’ response to the stress management question) was significantly more frequent among men than women (Table 3).

### 3.4. Factors Influencing Skin Signs and Symptoms

#### 3.4.1. Factors Influencing the Presence of Pruritus

**Figure 1:**
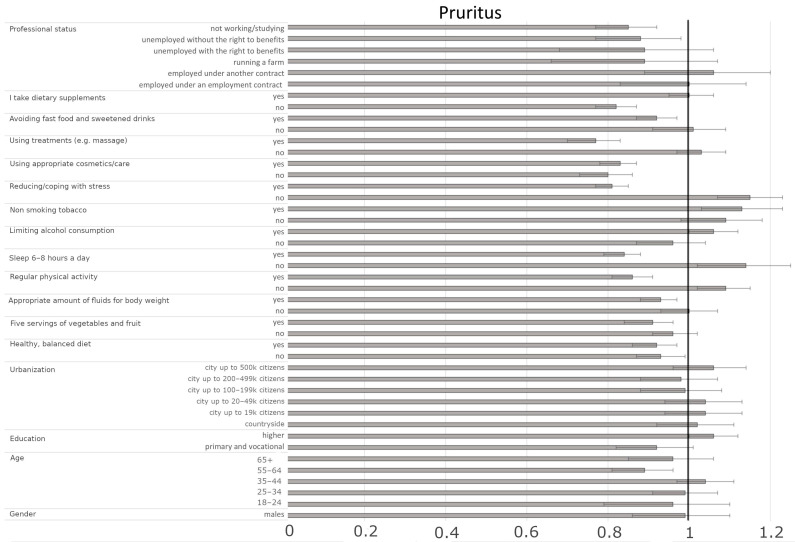
Odds ratios and 95% confidence intervals for the relationships between the presence of pruritus and sociodemographic and health-related factors across the entire group (the border between dark grey and bright grey shows the odds ratio of the parameter, the black lines show their 95% confidence intervals, and the vertical black line shows a reference value).

Considering sociodemographic factors, our study revealed that age and professional status influence the occurrence of pruritus. Older adults aged 55–64 years (OR = 0.89, CI95% [0.82–0.97], *p* = 0.0053) and unemployed individuals without benefits or those not working/students were less likely to experience pruritus (OR = 0.88, CI95% [0.78–0.99], *p* = 0.0319 and OR = 0.85, CI95% [0.78–0.93], *p* < 0.001, respectively). Diet and lifestyle factors, as indicators of well-being, seem to play a significant role. Consuming five servings of fruits and vegetables per day reduced the likelihood of pruritus by 9% (OR = 0.91, CI95% [0.86–0.98], *p* = 0.0075), while adequate fluid intake relative to body weight lowered the probability by 7% (OR = 0.93, CI95% [0.89–0.98], *p* = 0.0096). Additionally, participants who avoided fast food and sugary drinks had a lower likelihood of experiencing pruritus (OR = 0.92, CI95% [0.87–0.97], *p* = 0.0045). Engaging in regular physical activity was associated with a reduced probability of frequent pruritus (OR = 0.86, CI95% [0.81–0.91], *p* < 0.001), whereas physically inactive individuals had a higher likelihood (OR = 1.09, CI95% [1.03–1.16], *p* = 0.0049). Similarly, sleeping 6–8 h per night decreased the risk of pruritus (OR = 0.84, CI95% [0.80–0.89], *p* < 0.001). Interestingly, undergoing skincare treatments, such as massages, was also linked to a lower probability of pruritus (OR = 0.77, CI95% [0.71–0.84], *p* < 0.001). Moreover, stress management played a crucial role; individuals reporting ‘no’ to trying to reduce/deal with stress had a higher likelihood of experiencing pruritus (OR = 1.15, CI95% [1.07–1.23], *p* < 0.001), while those reporting ‘yes’ were less likely to exhibit pruritus (OR = 0.81, CI95% [0.77–0.85], *p* < 0.001, Figure 1, Table 4). These findings suggest that lifestyle habits and stress management play a significant role in the occurrence of pruritus, emphasizing the importance of holistic skin health management.

#### 3.4.2. Factors Influencing Burning Sensation of the Skin

**Figure 2:**
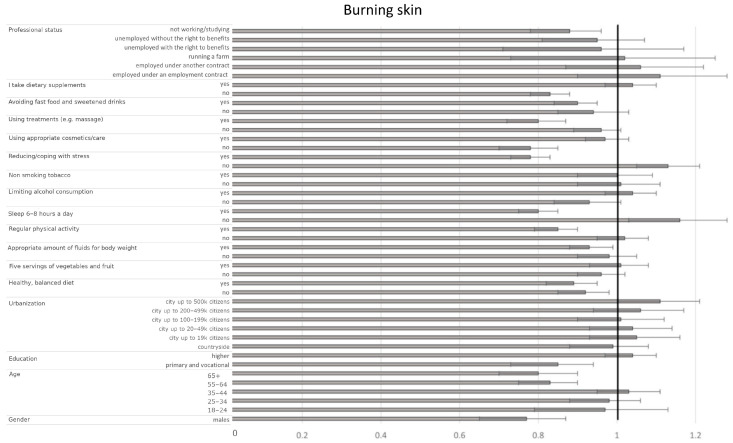
Odds ratios and 95% confidence intervals for the relationships between the presence of burning skin and sociodemographic and health-related factors across the entire group (the border between dark grey and bright grey shows the odds ratio of the parameter, the black lines show their 95% confidence intervals, and the vertical black line shows a reference value).

Among sociodemographic factors, men had a 23% lower likelihood of experiencing frequent burning sensations than women (OR = 0.77, CI95% [0.67–0.89], *p* < 0.001). Furthermore, individuals older than 55 years and those with primary or vocational education had a lower probability of experiencing burning skin sensation compared to the reference groups (45–54 years and secondary education, respectively) (e.g., 65+ vs. 45–54: OR = 0.80, CI95% [0.70–0.90], *p* < 0.001; primary/vocational vs. secondary: OR = 0.85, CI95% [0.76–0.97], *p* = 0.0118). Interestingly, residents of cities with populations exceeding 500,000 were more likely to experience burning skin sensations compared to those in cities of 50–99 k inhabitants (OR = 1.11, CI95% [1.01–1.22], *p* = 0.0390). Unemployed or studying individuals had a lower probability of experiencing burning skin sensations compared to pensioners (OR = 0.88, CI95% [0.80–0.98], *p* = 0.0168). Several well-being indicators were associated with reduced odds of burning sensations: proper hydration (yes vs. sometimes: OR = 0.93, CI95% [0.87–0.98], *p* = 0.0099), regular physical activity (yes vs. sometimes: OR = 0.85, CI95% [0.80–0.91], *p* < 0.001), and sleeping 6–8 h a night (yes vs. sometimes: OR = 0.80, CI95% [0.75–0.85], *p* < 0.001). Poor stress management (reporting ‘no’ vs. ‘sometimes’) increased the risk (OR = 1.13, CI95% [1.05–1.21], *p* = 0.0014), while effective stress management (reporting ‘yes’ vs. ‘sometimes’) was associated with a lower probability (OR = 0.78, CI95% [0.73–0.83], *p* < 0.001). Undergoing skincare treatments such as massages was also associated with reduced odds (yes vs. sometimes: OR = 0.80, CI95% [0.73–0.88], *p* < 0.001, Figure 2, Table 5).

#### 3.4.3. Factors Influencing the Presence of Skin Redness

**Figure 3:**
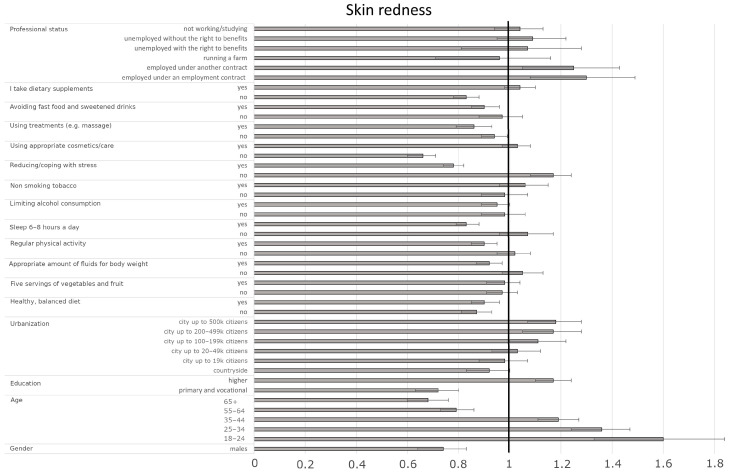
Odds ratios and 95% confidence intervals for the relationships between the presence of skin redness and sociodemographic and health-related factors across the entire group (the border between dark grey and bright grey shows the odds ratio of the parameter, the black lines show their 95% confidence intervals, and the vertical black line shows a reference value).

**Figure 4:**
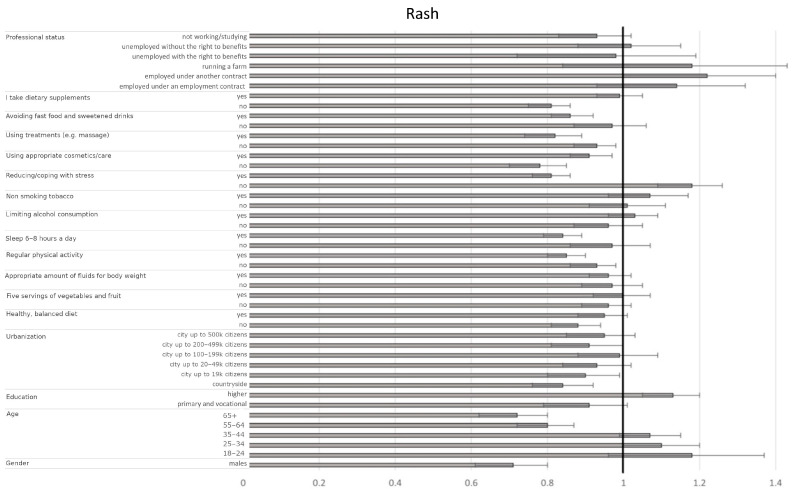
Odds ratios and 95% confidence intervals for the relationships between the presence of rash and sociodemographic and health-related factors across the entire group (the border between dark grey and bright grey shows the odds ratio of the parameter, the black lines show their 95% confidence intervals, and the vertical black line shows a reference value).

The most significant factors affecting skin redness included gender, age, education level, degree of urbanization, several well-being indicators (diet, hydration, physical activity, sleep, stress management, fast food avoidance, supplementation), cosmetic/treatment use, and professional status. Men had a 26% lower likelihood of experiencing frequent skin redness compared to women (OR = 0.74, CI95% [0.65–0.84], *p* < 0.001). Younger individuals (e.g., 18–24 vs. 45–54: OR = 1.60, CI95% [1.36–1.87], *p* < 0.001), those with higher education (vs. secondary: OR = 1.17, CI95% [1.10–1.24], *p* < 0.001), and residents of larger cities (e.g., ≥500 k vs. 50–99 k: OR = 1.18, CI95% [1.08–1.29], *p* < 0.001) were more likely to experience frequent skin redness than older individuals, those with lower education, and residents of smaller towns/rural areas (Figure 3, Table 6). Individuals employed under an employment contract or a civil law contract had a higher probability of frequent skin redness compared to pensioners (OR = 1.30, CI95% [1.11–1.52], *p* = 0.0010 and OR = 1.25, CI95% [1.07–1.45], *p* = 0.0038, respectively). Adherence to positive well-being indicators such as a healthy, balanced diet (yes vs. sometimes: OR = 0.90, CI95% [0.84–0.95], *p* < 0.001), adequate fluid intake (yes vs. sometimes: OR = 0.92, CI95% [0.87–0.97], *p* = 0.0017), regular physical activity (yes vs. sometimes: OR = 0.90, CI95% [0.85–0.95], *p* < 0.001), and sleeping 6–8 h per night (yes vs. sometimes: OR = 0.83, CI95% [0.78–0.87], *p* < 0.001) were associated with reduced odds of skin redness. Avoiding fast food/sugary drinks (yes vs. sometimes: OR = 0.90, CI95% [0.84–0.95], *p* < 0.001) and using treatments like massages (yes vs. sometimes: OR = 0.86, CI95% [0.79–0.93], *p* < 0.001) were also protective. Conversely, poor stress management (no vs. sometimes: OR = 1.17, CI95% [1.10–1.26], *p* < 0.001) was associated with increased odds, while effective stress management (yes vs. sometimes: OR = 0.78, CI95% [0.74–0.82], *p* < 0.001) was protective. Body weight also played a role; participants with higher body mass had an increased likelihood of experiencing skin redness (OR = 1.07 per unit increase, CI95% [1.01–1.13], *p* = 0.0212). Factors influencing skin rash were also comprehensively analyzed, with detailed predictors and their associations presented in Figure 4 and Table 7.

#### 3.4.4. Factors Influencing the Presence of Skin Desquamation

**Figure 5:**
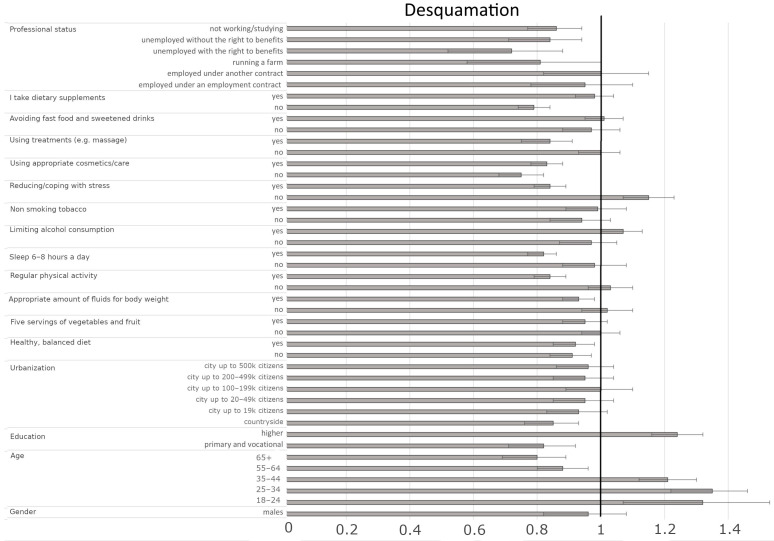
Odds ratios and 95% confidence intervals for the relationships between desquamation and sociodemographic and health-related factors across the entire group (the border between dark grey and bright grey shows the odds ratio of the parameter, the black lines show their 95% confidence intervals, and the vertical black line shows a reference value).

The occurrence of skin desquamation depends on several factors, including sociodemographic features (age, education, urbanization), well-being indicators (diet, hydration, physical activity, sleep, alcohol use, stress management, supplementation), cosmetic/treatment use, and professional status. Younger individuals (e.g., 25–34 vs. 45–54: OR = 1.35, CI95% [1.24–1.48], *p* < 0.001) and those with higher education (vs. secondary: OR = 1.24, CI95% [1.16–1.32], *p* < 0.001) were more likely to experience frequent skin desquamation than older individuals and those with primary/vocational education, respectively (Figure 5, Table 8). Interestingly, rural residents had lower odds of skin desquamation (vs. 50–99 k city: OR = 0.85, CI95% [0.77–0.94], *p* = 0.0021). Unemployed individuals and students were less likely to experience skin desquamation compared to pensioners. Regarding well-being indicators, adequate hydration (yes vs. sometimes: OR = 0.93, CI95% [0.88–0.98], *p* = 0.0108), regular physical activity (yes vs. sometimes: OR = 0.84, CI95% [0.79–0.89], *p* < 0.001), and adequate sleep (yes vs. sometimes: OR = 0.82, CI95% [0.78–0.87], *p* < 0.001) were associated with lower odds of desquamation. Stress management was crucial—those reporting ‘no’ (vs. ‘sometimes’) had higher odds (OR = 1.15, CI95% [1.07–1.23], *p* < 0.001), while those reporting ‘yes’ (vs. ‘sometimes’) had lower odds (OR = 0.84, CI95% [0.79–0.89], *p* < 0.001). Using appropriate cosmetics/care (yes vs. sometimes: OR = 0.83, CI95% [0.78–0.88], *p* < 0.001) and undergoing massages/other skincare treatments (yes vs. sometimes: OR = 0.84, CI95% [0.77–0.93], *p* < 0.001) also reduced the likelihood of skin desquamation.

#### 3.4.5. Factors Influencing the Presence of Sunburn

**Figure 6:**
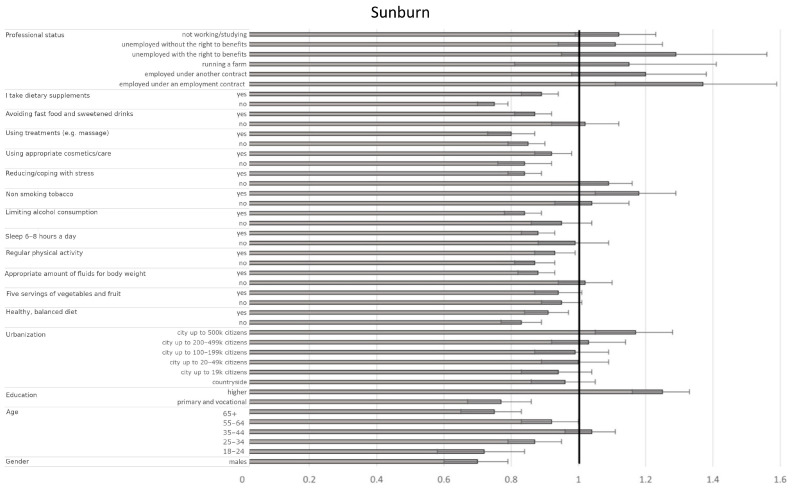
Odds ratios and 95% confidence intervals for the relationships between the presence of sunburn and sociodemographic and health-related factors across the entire group (the border between dark grey and bright grey shows the odds ratio of the parameter, the black lines show their 95% confidence intervals, and the vertical black line shows a reference value).

Regarding sociodemographic factors, men had a lower probability of frequent sunburns compared to women (OR = 0.70, CI95% [0.61–0.80], *p* < 0.001). Higher education levels were associated with an increased likelihood of sunburn compared to secondary education (OR = 1.25, CI95% [1.17–1.34], *p* < 0.001). Residents of cities with populations over 500,000 had higher odds of experiencing sunburn compared to those in 50–99 k cities (OR = 1.17, CI95% [1.06–1.29], *p* = 0.0016). Several well-being indicators were associated with lower odds of sunburns, including adequate fluid intake (yes vs. sometimes: OR = 0.88, CI95% [0.83–0.94], *p* < 0.001), sleeping 6–8 h per night (yes vs. sometimes: OR = 0.88, CI95% [0.83–0.93], *p* < 0.001), and severely limiting alcohol consumption (yes vs. sometimes: OR = 0.84, CI95% [0.79–0.90], *p* < 0.001). Avoiding fast food/sweetened drinks (yes vs. sometimes: OR = 0.87, CI95% [0.82–0.93], *p* < 0.001) and using treatments like massages (yes vs. sometimes: OR = 0.80, CI95% [0.73–0.87], *p* < 0.001) were also protective. Furthermore, stress management played a significant role, as poor stress management (no vs. sometimes) was linked to higher odds (OR = 1.09, CI95% [1.02–1.18], *p* = 0.0168), while effective stress management (yes vs. sometimes) was associated with lower odds (OR = 0.84, CI95% [0.79–0.89], *p* < 0.001). Higher body mass was also associated with an increased probability of experiencing sunburn (OR = 1.12 per unit increase, CI95% [1.06–1.20], *p* < 0.001, Figure 6, Table 9).

## 4. Discussion

Our study, analyzing data from over 27,000 Polish adults, revealed significant associations between self-reported stress management, various well-being indicators, sociodemographic factors, and the prevalence of common skin signs and symptoms. A key finding was the consistent link between stress management and all six investigated manifestations (pruritus, burning sensations, redness, rash, desquamation, and sunburn). Individuals reporting effective stress coping strategies related to skin health had significantly lower odds of experiencing these issues frequently, while those reporting an inability to manage stress (‘no’ response) consistently showed higher odds (Table 4, Table 5, Table 6, Table 7, Table 8 and Table 9). This underscores the profound connection between psychological state and cutaneous health, supporting the growing body of evidence on the psychodermatological axis [8,9,10]. Our findings, specifically demonstrating this link for a range of common, self-reported symptoms in a large population, extend previous research that has often focused on specific, clinically diagnosed dermatoses. For example, while Al’Abadie et al. [3] identified stress as a precursor to psoriasis onset and exacerbation, our study suggests that even more prevalent, non-specific symptoms like pruritus and redness are similarly associated with perceived stress coping abilities. This broadens the potential relevance of psychosocial interventions beyond specialized dermatological conditions to more common skin complaints.

Nevertheless, several limitations should be acknowledged. First, the assessment of stress management and well-being indicators relied on single-item, self-report questions rather than validated psychometric scales (e.g., PSS, DLQI, WHO-5). While facilitating participation in a large-scale survey, this approach limits the depth and precision of these psychosocial assessments and precludes direct comparison with studies using standardized instruments. Future research could benefit from incorporating validated questionnaires to provide a more robust evaluation of stress and well-being. Given that the key psychosocial predictor—‘stress coping ability’ related to skin health—was assessed via a single, self-reported item specifically phrased for this survey (‘Do you try to reduce/deal with stress to take care of your skin?’) and not a standardized, validated psychometric scale for general stress or coping, its statistical associations, though consistently observed across the six dermatological symptom models, must be interpreted with particular caution. The subjective nature of this measure means that its relationship with skin symptoms reflects perceived coping efforts in the context of skin health rather than a clinically validated stress metric. Seventh, when interpreting the results, it is essential to distinguish between statistical significance and potential clinical relevance. Due to the study’s very large sample size (N = 27,000), even modest associations can achieve statistical significance (*p* ≤ 0.05). For instance, several odds ratios reported, while statistically significant, are close to 1.0 (e.g., in the range of 0.90–1.10). Such ORs indicate a statistically reliable but potentially small effect size. While these findings contribute to understanding population-level associations, their direct clinical impact on an individual patient might be limited. Therefore, the emphasis should be on consistent patterns and the magnitude of effects when considering potential clinical implications, rather than solely on statistical significance, particularly for those associations with odds ratios proximal to null. Furthermore, it is important to note that while the ‘National Healthy Skin Test’ questionnaire underwent development and pilot testing by its creators for its primary purpose as a broad public health survey, the instrument itself, including the specific questions on dermatological symptoms and lifestyle factors as utilized in our secondary analysis, was not subjected to an additional formal psychometric validation study (e.g., test-retest reliability, convergent/divergent validity) within the direct context of this specific research paper. The questions were designed for broad population screening, and while the self-reported skin symptoms are common and generally understood, this lack of a dedicated validation for our analytical purposes is a limitation. This reinforces the reliance on self-perceived data and the potential for associated biases, as previously noted. Second, our reliance on self-reported data for all variables, including the presence and frequency of skin manifestations, represents another methodological consideration. While enabling large-scale assessment, self-reports may be influenced by recall bias or varying symptom perception and recognition thresholds among participants. Objective dermatological examinations were not performed. Consequently, the findings of this study primarily relate to associations with these common, self-perceived skin issues, and the term ‘dermatological manifestations’ used herein refers to these self-reported symptoms. The results should be interpreted with caution in this context. The study does not allow for conclusions about specific, clinically diagnosed dermatological diseases, nor can it establish causality given its cross-sectional design. Generalizing these findings to broader clinical dermatological conditions or to infer direct health implications requires further research employing validated, multi-item psychometric instruments for stress and well-being, alongside objective dermatological evaluations and clinical diagnoses. Such research would be essential to corroborate and expand upon these exploratory findings. Third, the potential overlap between subjective terms like ‘rash’ and ‘redness’, although analyzed separately, could reflect variability in participant interpretation. Fourth, the cross-sectional design precludes establishing causality; we can only report associations. It is also important to acknowledge the likely bidirectional relationship between stress and skin symptoms; while stress may exacerbate skin manifestations, as suggested by our findings, the presence of bothersome skin symptoms can, in turn, contribute to increased psychological distress, a phenomenon well-documented in psychodermatology [5]. Our study design does not allow us to disentangle this complex reciprocal influence. Fifth, our study did not collect data on, nor control for, several potentially important variables such as genetic predispositions to skin conditions or stress sensitivity, nor did we have information on pre-existing, clinically diagnosed dermatological diseases among participants. Such unmeasured factors could act as confounders or effect modifiers in the observed relationships between psychosocial factors and self-reported skin symptoms, and their absence from our models is a limitation. Sixth, our analytical approach involved conducting separate ordinal logistic regression models for each of the six self-reported dermatological manifestations. While this allowed for a detailed investigation of predictors for each specific symptom, testing multiple hypotheses across these models (each including numerous predictors) without formal correction for multiple comparisons (e.g., Bonferroni correction or controlling the False Discovery Rate) increases the potential for Type I errors (false positives). Although the consistency of some findings across several symptom models (such as the association with stress management) may suggest a degree of robustness, the statistical significance of individual predictors should be interpreted with this consideration in mind. Future studies might consider multivariate response models or apply appropriate *p*-value adjustments if similar exploratory analyses across multiple endpoints are undertaken. Finally, while the large sample size is a strength, several sampling characteristics and methodological choices impact the generalizability of our findings. Recruitment was conducted via Medonet, a major online platform, which, while facilitating access to a large and diverse adult cohort, inherently introduces selection bias. This approach may underrepresent individuals without consistent internet access or those with lower digital literacy, and their experiences with skin manifestations and psychosocial factors may differ. Furthermore, our study population exhibited a significant gender imbalance, with a predominance of female respondents (81.7% women, 18.1% men). While gender was accounted for in our statistical analyses, this skew means that findings, particularly those not specifically stratified by gender in all aspects, should be generalized with caution, especially to the male segment of the Polish population or when comparing to national demographic distributions. Additionally, the study was designed to focus on the adult population (≥18 years old), and therefore, our results are not representative of children and adolescents. Consequently, the conclusions drawn should be considered primarily applicable to the adult, online Polish population who participated, with a clear female predominance. Caution is advised when extrapolating these findings to underrepresented or excluded demographic groups, and further research in those populations would be valuable.

Before discussing these factors in detail, it is important to clarify the conceptual distinction maintained in our study between ‘self-reported stress management related to skin health’ and the ‘lifestyle factors’ (operationalized as indicators of well-being). While these psychosocial constructs are understood to be interconnected and may exert reciprocal influences in daily life—for instance, poor lifestyle choices can potentially exacerbate physiological stress responses, and conversely, chronic stress can negatively impact lifestyle behaviors—our study investigated them as distinct predictors of the reported dermatological symptoms. The ‘stress management’ variable, derived from the question ‘Do you try to reduce/deal with stress to take care of your skin?’, specifically reflects participants’ self-reported active efforts in this regard. In contrast, the ‘well-being indicators’ capture adherence to certain health-promoting lifestyle practices, which, while contributing to overall well-being and potentially buffering or interacting with stress, were analyzed as separate variables in our models.

One of the determinants of the presence of skin manifestations appears to be lifestyle factors indicative of well-being. Our study operationalized well-being through factors such as healthy diet, adequate hydration, sufficient sleep, regular physical activity, limited alcohol use, non-smoking status, and avoidance of fast food. We found that maintaining positive lifestyle habits was generally associated with a decreased likelihood of suffering from the studied dermatological signs and symptoms. For instance, sleeping for 6–8 h per night was associated with lower odds of experiencing pruritus, burning sensations, redness, rash, desquamation, and sunburn. This aligns with research showing sleep deprivation can trigger inflammation, increase cortisol, and weaken the skin barrier [9]. Our population-based findings reinforce the importance of adequate sleep not just for general health, but specifically in relation to a wide spectrum of common self-perceived skin issues, an aspect that may be underemphasized in routine patient advice for skin health. Similarly, regular physical activity (at least 150 min per week) was associated with decreased odds of pruritus, burning sensations, redness, and desquamation, likely through improved circulation, nutrient delivery, and stress reduction [11]. Adherence to a healthy diet and adequate hydration also showed protective associations against several symptoms, emphasizing the holistic nature of skin health. Mechanistically, these lifestyle factors influence skin barrier function, inflammatory pathways, and oxidative stress levels, thereby modulating susceptibility to common skin complaints [12,13].

The impact of stress and well-being indicators on skin manifestations varied across sociodemographic groups. Our study demonstrated significant gender differences for most self-reported skin symptoms. Men were less likely to report frequent burning sensations and redness but more likely to report frequent rash, desquamation, and sunburn. The lack of a significant gender difference for pruritus prevalence, in contrast, might suggest distinct underlying mechanisms or reporting patterns for this particular symptom compared to the others investigated. These observed gender-based variations likely reflect a complex interplay of factors. Biologically, differences in skin physiology, such as average skin thickness, sebum production, and hormonal fluctuations (e.g., estrogen, androgens), are known to influence skin barrier function, sensitivity, and inflammatory responses, potentially contributing to varied symptom experiences. Psychosocially and behaviorally, there may be gender differences in the perception and reporting of symptoms, health-seeking behaviors, exposure to environmental or occupational irritants, and lifestyle choices, including skincare and cosmetic use [14]. For instance, women might use a wider array of topical products, potentially increasing the risk of irritation or allergic reactions for some, or they may exhibit greater health awareness, leading to higher reporting rates of certain symptoms. Conversely, men might engage more frequently in activities leading to physical skin insults or have different patterns of sun exposure, contributing to higher reports of sunburn or desquamation in our cohort. A deeper understanding of these multifaceted influences warrants further specific investigation to disentangle the precise contributions of biological sex versus gendered societal and behavioral factors.

Age also played a significant role. Younger adults (18–34 years) were generally more likely to report redness, rash, and desquamation, whereas older adults (>55 years) reported less pruritus and burning sensation. The higher prevalence of rash-like symptoms in younger individuals could potentially reflect conditions more common in this age group, such as flares of atopic conditions or inflammatory responses [15,16], although our study did not assess specific diagnoses.

Interestingly, higher education levels were associated with an increased likelihood of reporting sunburn, rash, desquamation, and redness. This might be linked to lifestyle factors (e.g., more outdoor recreation, travel), occupational factors, or increased cosmetic use [17]. Alternatively, regarding the term ‘rash’, individuals with higher education might possess greater health literacy or awareness, leading to increased recognition and reporting of subtle skin changes. This highlights an important consideration when interpreting self-reported data across different educational backgrounds. Similarly, residence in large urban centers was associated with increased odds of sunburn, redness, and burning sensations. This aligns with studies suggesting urban environments may exacerbate certain skin issues, potentially due to higher levels of air pollution (e.g., ozone, particulate matter), which can induce oxidative stress and inflammation in the skin [18]. While some epidemiological studies, including in Poland, have shown higher rates of allergic diseases like asthma or rhinitis in urban versus rural settings [19,20], the picture for general skin symptoms can be complex, with allergen profiles and lifestyle factors also differing significantly between environments [21]. Our findings contribute to the understanding of how urban living might influence common skin complaints beyond specific allergic diagnoses. Understanding these interactions between sociodemographics, environment, stress, and well-being is essential for tailoring interventions.

Comparing our findings with international data provides further context. For instance, the overall prevalence of any pruritus (defined as itch in the last 7 days) was recently reported to be around 35.9% in Europe in a large multinational study, with higher rates in older adults and women [22]. While our study used different frequency categories (‘occasional’ or ‘frequent’), the substantial proportion of our Polish cohort reporting at least occasional pruritus (approx. 65.5% overall, calculated from Table 2) appears high relative to this European benchmark, although methodological differences in assessment preclude direct comparison. Studies focusing specifically on chronic pruritus (lasting >6 weeks) report lower prevalence rates in the general European population, typically ranging from 13.5% (point prevalence) to 22% (lifetime prevalence) [23,24]. Further research using standardized definitions and timeframes is needed to accurately compare the burden of common skin symptoms across different populations.

The findings strongly support the integration of psychosocial assessment and management into dermatological practice. The consistent association between poor stress management and increased frequency of common skin signs and symptoms highlights a potential target for intervention. The associations observed in our study might suggest potential value for healthcare professionals to consider inquiring about perceived stress levels and coping mechanisms, particularly when patients present with persistent or recurrent self-reported common skin complaints such as pruritus, redness, or rash. However, the clinical utility and impact of such inquiries would require validation through further dedicated research. Based on our findings regarding common self-reported symptoms, it could be hypothesized that educating patients about the potential stress-skin connection and exploring appropriate stress-reduction techniques might offer a complementary approach to traditional dermatological therapies for these issues. However, the efficacy and applicability of such interventions need to be confirmed in dedicated clinical trials [10]. Further research exploring the efficacy of specific stress-reduction interventions for these common skin symptoms in diverse populations is warranted.

Exploring the integration of psychodermatological approaches may offer potential avenues for benefiting patients and healthcare systems in the context of common skin symptoms. While further research is essential to validate specific interventions, exploring the integration of psychodermatological approaches may offer potential avenues for benefiting patients with common, self-reported skin symptoms. Based on our findings and the broader literature, potential components of a comprehensive strategy in routine clinical practice could be considered for future development and testing. For instance, this might involve incorporating brief, validated screening questions for perceived stress levels and key well-being indicators during dermatological consultations, particularly for patients with recurrent symptoms. Furthermore, developing and providing accessible patient education materials that explain the interplay between stress, lifestyle, and common skin symptoms could empower patients with basic self-help information. Dermatological settings could also explore training for staff to offer brief, empathetic counseling or motivational interviewing focused on acknowledging patients’ stress and encouraging simple management strategies. Where psychosocial needs exceed the scope of dermatological practice, establishing clearer referral pathways to psychological or specialized lifestyle support services would be crucial. Finally, contributing to broader public health campaigns that raise awareness about the holistic nature of skin health, including the impact of stress and well-being, could also be beneficial. Further investigation is needed to determine if such approaches could improve clinical outcomes, enhance treatment adherence, or potentially reduce healthcare costs, thereby promoting a more holistic, patient-centered model of care for these common complaints. Our findings provide strong evidence supporting a biopsychosocial model that recognizes common skin manifestations not merely as isolated physical issues, but as complex phenomena influenced by psychological state, lifestyle factors, and sociodemographic context. Finally, it is crucial that the findings of this study are not oversimplified. While our results highlight significant associations between psychosocial factors and the prevalence of common, self-reported skin symptoms in a large population sample, they do not imply that all dermatological conditions are solely attributable to stress, nor should these findings be interpreted as diminishing the importance of thorough dermatological evaluation and appropriate medical management for any skin concern. The associations observed are part of a complex interplay of factors influencing skin health.

## 5. Conclusions

This large-scale cross-sectional study in a Polish adult population identified significant associations between self-reported stress management, various lifestyle/well-being indicators, sociodemographic factors, and the prevalence of six common, self-reported dermatological manifestations. Our findings lend support to a biopsychosocial model for understanding these common skin symptoms, suggesting that psychological state, lifestyle choices, and sociodemographic context are interlinked with individuals’ perceptions of their skin health.

It is crucial that these findings are not oversimplified. While this study highlights these associations, it does not imply that all dermatological conditions are solely attributable to stress, nor do these findings diminish the importance of comprehensive dermatological evaluation and medical management for any skin concern. The associations observed are part of a complex interplay of factors.

Given the study’s limitations, including its cross-sectional design and reliance on self-reported data, a clear forward-looking perspective is essential. Future longitudinal studies are needed to confirm the observed associations, explore potential causal pathways, and more definitively assess the directionality of these relationships. Such research would greatly benefit from the incorporation of validated, multi-item psychometric scales for psychosocial assessments and objective clinical evaluations of skin manifestations, moving beyond self-report. Furthermore, prospective studies could investigate the efficacy of specific, targeted interventions, such as tailored stress-management programs or lifestyle modification support, for individuals frequently affected by these common skin symptoms. Additionally, future analyses could explore potential effect modification by key sociodemographic variables, such as age or professional status, to understand if the observed associations between psychosocial factors and skin symptoms differ across these subgroups. Exploring the mechanisms underlying the observed sociodemographic variations in both symptom reporting and their psychosocial correlates also presents a valuable avenue for future inquiry. Ultimately, continued research in this area holds the potential to inform more holistic public health strategies and enhance patient-centered care for common dermatological complaints by better integrating psychosocial awareness into their understanding and management.

## Figures and Tables

**Table 1:** Demographic data of the respondent diversified by gender (the numbers (n) and frequencies (%) of the study population with their sociodemographic characteristics; total: 27,000).

Trait	Men n (%)	Women n (%)	Other n (%)	Total n (%)	Men-Women Difference Significance
**Gender**	4887 (18.1)	22,043 (81.7)	70 (0.3)	27,000 (100.0)	
**AGE (years)**					***χ* ^2^ = 382.47;** ***df * = 5;** ***p* < 0.001**
18–24	172 (0.6)	770 (2.9)	13 (0.05)	955 (3.5)	
25–34	658 (2.4)	3061 (11.3) **^s^**	9 (0.03)	3728 (13.8)	
35–44	987 (3.7)	5312 (19.7) **^s^**	13 (0.05)	6312 (23.4)	
45–54	849 (3.1)	5530 (20.5) **^s^**	17 (0.1)	6396 (23.7)	
55–64	864 (3.2) **^s^**	3653 (13.5)	5 (0.02)	4522 (16.7)	
65+	1357 (5.0) **^s^**	3717 (13.8)	13 (0.05)	5087 (18.8)	
**Education**					***χ* ^2^ = 46.97;** ***df * = 2;** ***p* < 0.001**
primary and vocational	562 (2.1) **^s^**	1853 (6.9)	13 (0.05)	2428 (9.0)	
secondary and post-secondary	1755 (6.5)	8149 (30.2) **^s^**	31 (0.1)	9935 (36.8)	
higher	2570 (9.5)	12,041 (44.6) **^s^**	26 (0.1)	14,637 (54.2)	
**Urbanization**					***χ* ^2^ = 113.46** ***df * = 6** ***p* < 0.001**
countryside	861 (3.2)	4609 (17.1) **^s^**	17 (0.1)	5487 (20.3)	
city up to 19,000 citizens	547 (2.0)	2538 (9.4) **^s^**	5 (0.02)	3090 (11.4)	
city of 20–49,000 citizens	619 (2.3)	3517 (13.0) **^s^**	1 (0.004)	4137 (15.3)	
city of 50–99,000 citizens	558 (2.1) **^s^**	2382 (8.8)	7 (0.03)	2947 (10.9)	
city of 100–199,000 citizens	571 (2.1) **^s^**	2162 (8.0)	7 (0.03)	2740 (10.1)	
city of 200–499,000 citizens	528 (2.0)	2497 (9.2) **^s^**	11 (0.04)	3036	
city of 500,000 or more citizens	1203 (4.5) **^s^**	4338 (16.1)	22 (0.1)	5563 (20.6)	
**Professional Status**					***χ* ^2^ = 125.44** ***df * = 6** ***p* < 0.001**
Employed under an employment contract or running your own business	2805 (10.4)	13,265 (49.1) **^s^**	34 (0.1)	16,104 (59.6)	
Employed under another contract (mandate contract. contract for specific work)	314 (1.2)	1381 (5.1)	4 (0.01)	1699 (6.3)	
Running a farm	60 (0.2)	296 (1.1)	1 (0.004)	357 (1.3)	
Pensioner	1437 (5.3) **^s^**	5121 (19.0)	15 (0.1)	6573 (24.3)	
Unemployed with the right to benefits	32 (0.1)	263 (1.0) **^s^**	1 (0.004%)	296 (1.1)	
Unemployed without the right to benefits	118 (0.4)	813 (3.0) **^s^**	6 (0.02)	937 (3.5)	
Not working/studying	121 (0.4)	904 (3.3) **^s^**	9 (0.03)	1034 (3.8)	

**^s^** Surplus—value significantly greater than expected (separately for each category). The term ‘in red’ is bolded for emphasis.

**Table 2:** The frequency of skin signs and symptoms among the tested responders, diversified by gender (total: 27,000).

Trait	Men	Women	Other	Total	Men-Women Difference Significance
**Pruritus**					*χ*^2^ = 5.86; *df* = 4; *p* = 0.2098
at least several times a month	1216	5221	21	6458	
%	4.5%	19.3%	0.1%	23.9%	
occasionally—less than a few times a month	1977	9233	27	11,237	
%	7.3%	34.2%	0.1%	41.6%	
never	1694	7589	22	9305	
%	6.3%	28.1%	0.1%	34.5%	
**Burning sensation**					***χ* ^2^ = 97.52; *df* = 4;** ***p* < 0.001**
at least several times a month	390	2000 **^s^**	11	2401	
%	1.4%	7.4%	0.04%	8.9%	
occasionally—less than a few times a month	1018	5919 **^s^**	20	6957	
%	3.8%	21.9%	0.1%	25.8%	
never	3479 **^s^**	14,124	39	17,642	
%	12.9%	52.3%	0.1%	65.3%	
**Redness**					***χ*^2^ = 217.59;** ***df * = 4;** ***p * < 0.001**
at least several times a month	862	4756 **^s^**	17	5635	
%	3.2%	17.6%	0.1%	20.9%	
occasionally—less than a few times a month	1476	8328 **^s^**	23	9827	
%	5.5%	30.8%	0.1%	36.4%	
never	2549 **^s^**	8959	30	11,538	
%	9.4%	33.2%	0.1%	42.7%	
**Rash**					***χ^2^ * = 151.04; * df * = 4;** ***p < * 0.001**
at least several times a month	351 **^s^**	1424	5	1780	
%	1.3%	5.3%	0.02%	6.6%	
occasionally—less than a few times a month	1121	7011 **^s^**	26	8158 **^s^**	
%	4.15%	26.0%	0.1%	30.2%	
never	3415 **^s^**	13,608	39	17,062	
%	12.7%	50.4%	0.1%	63.2%	
**Desquamation**					***χ^2^ = * 110.88; * df = * 4;** ***p * < 0.001**
at least several times a month	660 **^s^**	2463	16	3139	
%	2.4%	9.1%	0.1%	11.6%	
occasionally—less than a few times a month	962	5802 **^s^**	20	6784	
%	3.6%	21.5%	0.1%	25.1%	
never	3265 **^s^**	13,778	34	17,077	
%	12.1%	51.0%	0.1%	63.3%	
**Sunburn**					***χ* ^2^ = 63.71; *df* = 4;** ***p* < 0.001**
at least several times a month	104 **^s^**	393	2	499	
%	0.4%	1.5%	0.01%	1.9%	
occasionally—less than a few times a month	1499	8067 **^s^**	20	9586	
%	5.6%	29.9%	0.07%	35.5%	
never	3284 **^s^**	13,583	48 **^s^**	16,915	
%	12.2%	50.3%	0.2%	62.7%	

**^s^** Surplus—value significantly greater than expected (separately for each category). The term ‘in red’ is bolded for emphasis.

**Table 3:** The presence of features characterizing the skin conditions among studied responders diversified by gender (total: 27,000).

Trait	Men	Women	Other	Total	Men-Women Difference Significance
**Freckles**					***χ* ^2^ = 327.73; *df* = 2;** ***p* < 0.001**
yes	797	6245 **^s^**	16	7058	
%	3.0%	23.1%	0.1%	26.1%	
no	3789 **^s^**	14,962	48	18,799	
%	14.0%	55.4%	0.2%	69.6%	
don’t know	301 **^s^**	836	6	1143	
%	1.1%	3.1%	0.02%	4.2%	
**Moles/birthmarks**					***χ* ^2 ^ = 133.29; *df* = 2;** ***p* < 0.001**
yes	3182	16,089 **^s^**	47	19,318	
%	11.8%	59.6%	0.2%	71.5%	
no	1422 **^s^**	5144	13	6579	
%	5.3%	19.1%	0.05%	24.4%	
don’t know	283 **^s^**	810	10	1103	
%	1.0%	3.0%	0.04%	4.1%	
**Discoloration/sunspots**					***χ* ^2^ = 884.71; *df* = 2;** ***p* < 0.001**
yes	981	9477 **^s^**	22	10,480	
%	3.6%	35.1%	0.1%	38.8%	
no	3386 **^s^**	10,911	37	14,334	
%	12.5%	40.4%	0.1%	53.1%	
don’t know	520 **^s^**	1655	11	2186	
%	1.9%	6.1%	0.04%	8.1%	
**Visible blood vessels**					***χ* ^2^ = 1187.95; *df* = 2;** ***p* < 0.001**
yes	1227	11,531 **^s^**	32	12,790	
%	4.5%	42.7%	0.1%	47.4%	
no	3210 **^s^**	9176	29	12,415	
%	11.9%	34.0%	0.1%	46.0%	
don’t know	450 **^s^**	1336	9	1795	
%	1.7%	4.9%	0.03%	6.6%	
**Dry skin (also if it only affects parts of it)**					***χ* ^2^ = 641.15; *df* = 2;** ***p* < 0.001**
yes	2248	14,423 **^s^**	49	16,720	
%	8.3%	53.4%	0.2%	61.93%	
no	2062 **^s^**	5902	14	7978	
%	7.6%	21.9%	0.1%	29.55%	
don’t know	577 **^s^**	1718	7	2302	
%	2.1%	6.4%	0.03%	8.53%	
**Oily skin (also if it only affects parts of it)**					***χ* ^2^ = 159.21; *df* = 2;** ***p* < 0.001**
yes	1486	8455 **^s^**	31	9972	
%	5.5%	31.3%	0.1%	36.9%	
no	2769 **^s^**	11,698	29	14,496	
%	10.3%	43.3%	0.1%	53.7%	
don’t know	632 **^s^**	1890	10	2532	
%	2.3%	7.0%	0.04%	9.4%	
**Delicate wrinkles**					***χ* ^2 ^ = 1099.32; *df* = 2;** ***p* < 0.001**
yes	2969	18,139 **^s^**	44	21,152	
%	11.0%	67.2%	0.2%	78.3%	
no	1471 **^s^**	3072	21	4564	
%	5.4%	11.4%	0.1%	16.9%	
don’t know	447 **^s^**	832	5	1284	
%	1.7%	3.1%	0.02%	4.8%	
**Crow’s feet**					***χ* ^2 ^ = 1428.80; *df* = 2;** ***p* < 0.001**
yes	1086	11,406	28	12,520	
%	4.0%	42.2%	0.1%	46.4%	
no	3136 **^s^**	9104	33	12,273	
%	11.6%	33.7%	0.1%	45.5%	
don’t know	665 **^s^**	1533	9	2207	
%	2.5%	5.7%	0.03%	8.2%	
**Deep wrinkles**					***χ* ^2^ = 151.70; *df* = 2;** ***p* < 0.001**
yes	421	3396 **^s^**	11	3828	
%	1.6%	12.6%	0.0%	14.2%	
no	3898 **^s^**	16,258	51	20,207	
%	14.4%	60.2%	0.2%	74.8%	
don’t know	568 **^s^**	2389	8	2965	
%	2.1%	8.8%	0.03%	11.0%	
**Glabellar lines** (Lion’s frown)					***χ* ^2^ = 1510.76; *df* = 2;** ***p* < 0.001**
yes	385	7907 **^s^**	19	8311	
%	1.4%	29.3%	0.1%	30.8%	
no	3127 **^s^**	10,431	35	13,593	
%	11.6%	38.6%	0.1%	50.3%	
don’t know	1375 **^s^**	3705	16	5096	
%	5.1%	13.7%	0.1%	18.9%	
**Breakouts**					***χ* ^2^ = 163.70; *df* = 2;** ***p* < 0.001**
yes	1350	8044 **^s^**	31	9425	
%	5.0%	29.8%	0.1%	34.9%	
no	3088 **^s^**	12,611	33	15,732	
%	11.4%	46.7%	0.1%	58.3%	
don’t know	449 **^s^**	1388	6	1843	
%	1.7%	5.1%	0.02%	6.8%	
**High sensitivity to external factors/cosmetics**					***χ* ^2^ = 693.64; *df* = 2;** ***p* < 0.001**
yes	720	7467 **^s^**	29	8216	
%	2.7%	27.7%	0.1%	30.4%	
no	3285 **^s^**	11,396	37	14,718	
%	12.2%	42.2%	0.1%	54.5%	
don’t know	882 **^s^**	3180	4	4066	
%	3.3%	11.8%	0.01%	15.1%	
**Furrows**					***χ* ^2 ^ = 255.05; *df* = 2;** ***p* < 0.001**
yes	583	4864 **^s^**	9	5456	
%	2.2%	18.0%	0.0%	20.2%	
no	3576 **^s^**	14,212	50	17,838	
%	13.2%	52.6%	0.2%	66.1%	
don’t know	728 **^s^**	2967	11	3706	
%	2.7%	11.0%	0.04%	13.7%	
**Drooping face oval**					***χ* ^2^ = 1898.490; *df* = 2;** ***p* < 0.001**
yes	492	9414 **^s^**	27	9933	
%	1.8%	34.9%	0.1%	36.8%	
no	3379 **^s^**	9006	31	12,416	
%	12.5%	33.4%	0.1%	46.0%	
don’t know	1016 **^s^**	3623	12	4651	
%	3.8%	13.4%	0.04%	17.2%	
**Loose skin**					***χ* ^2^ = 1080.79; *df* = 2;** ***p* < 0.001**
yes	576	7689 **^s^**	23	8288	
%	2.1%	28.5%	0.1%	30.7%	
no	3462 **^s^**	10,665	29	14,156	
%	12.8%	39.5%	0.1%	52.4%	
don’t know	849 **^s^**	3689	18	4556	
%	3.1%	13.7%	0.1%	16.9%	
**Erythema/redness**					***χ* ^2 ^ = 133.62; *df* = 2;** ***p* < 0.001**
yes	816	5336 **^s^**	18	6170	
%	3.0%	19.8%	0.1%	22.9%	
no	3543 **^s^**	14,758	39	18,340	
%	13.1%	54.7%	0.1%	67.9%	
don’t know	528 **^s^**	1949	13	2490	
%	2.0%	7.2%	0.05%	9.2%	
**Transverse forehead wrinkles**					***χ* ^2^ = 60.36; *df* = 2;** ***p* < 0.001**
yes	2091	9956 **^s^**	28	12,075	
%	7.7%	36.9%	0.1%	44.7%	
no	2285	10,501 **^s^**	35	12,821	
%	8.5%	38.9%	0.1%	47.5%	
don’t know	511 **^s^**	1586	7	2104	
%	1.9%	5.9%	0.03%	7.8%	
**Stretch marks**					***χ* ^2^ = 1775.03; *df* = 2;** ***p* < 0.001**
yes	737	10,568 **^s^**	31	11,336	
%	2.7%	39.1%	0.1%	42.0%	
no	3750 **^s^**	10,433	35	14,218	
%	13.9%	38.6%	0.1%	52.7%	
don’t know	400 **^s^**	1042	4	1446	
%	1.5%	3.9%	0.01%	5.4%	
**Cellulite**					***χ* ^2 ^ = 6042.44; *df* = 2;** ***p* < 0.001**
yes	283	14,452 **^s^**	33	14,768	
%	1.0%	53.5%	0.1%	54.7%	
no	4085 **^s^**	6033	30	10,148	
%	15.1%	22.3%	0.1%	37.6%	
don’t know	519 **^s^**	1558	7	2084	
%	1.9%	5.8%	0.03%	7.7%	
**Excessive body hair**					***χ* ^2^ = 86.14; *df* = 2;** ***p* < 0.001**
yes	616	3995 **^s^**	10	4621	
%	2.3%	14.8%	0.04%	17.1%	
no	3925 **^s^**	16,629	50	20,604	
%	14.5%	61.6%	0.2%	76.3%	
don’t know	346 **^s^**	1419	10	1775	
%	1.3%	5.3%	0.04%	6.6%	
Reducing/Coping with Stress					
**I try to reduce/deal with stress to take care of my skin.**					***χ* ^2^ = 97.08; *df* = 2;** ***p* < 0.001**
yes	2187 **^s^**	8263	28	10,478	
%	8.1%	30.6%	0.1%	38.8%	
sometimes	1906	10,111 **^s^**	28	12,045	
%	7.1%	37.4%	0.1%	44.6%	
no	794	3669 **^s^**	14	4477	
%	2.9%	13.6%	0.1%	16.6%	

**^s^** Surplus—value significantly greater than expected (separately for each category). The term ‘in red’ is bolded for emphasis.

**Table 4:** Results of logistic regression regarding sociodemographic and health-related factors associated with the presence of pruritus, as well as the odds ratio (OR) and its 95% confidence interval (CI) (in red: the most statistically significant (*p*-value < 0.05) predictors of the presence of pruritus). The term ‘in red’ is bolded for emphasis.

Trait	Estimate	OR	Lower 95 CI OR	Uppper 95 CI OR	*p*
**Intercept 1**	**−0.72**	**0.49**	**0.41**	**0.58**	**<0.001**
**Intercept 2**	**1.12**	**3.08**	**2.58**	**3.66**	**<0.001**
**Gender**					
men	−0.01	0.99	0.88	1.12	0.9069
women	reference				
**Age**					
18–24	−0.04	0.96	0.82	1.13	0.6110
25–34	−0.01	0.99	0.91	1.07	0.7798
35–44	0.04	1.04	0.97	1.11	0.2720
45–54	reference				
55–64	**−0.12**	**0.89**	**0.82**	**0.97**	**0.0053**
65+	−0.04	0.96	0.86	1.07	0.4742
**Education**					
primary and vocational	−0.08	0.92	0.83	1.02	0.1235
secondary and post-secondary	reference				
higher	0.06	1.06	1.00	1.12	0.0596
**Urbanization**					
countryside	0.02	1.02	0.93	1.12	0.6764
city up to 19,000 citizens	0.04	1.04	0.95	1.14	0.4066
city of 20–49,000 citizens	0.04	1.04	0.95	1.14	0.3801
city of 50–99,000 citizens	reference				
city of 100–199,000 citizens	−0.01	0.99	0.90	1.10	0.8982
city of 200–499,000 citizens	−0.02	0.98	0.89	1.08	0.6734
city of to 500,000 or more citizens	0.06	1.06	0.98	1.16	0.1587
**A healthy, balanced diet**					
no	**−0.08**	**0.93**	**0.87**	**0.99**	**0.0232**
sometimes	reference				
yes	**−0.08**	**0.92**	**0.87**	**0.98**	**0.0099**
**At least five servings of vegetables and fruit**					
no	−0.05	0.96	0.90	1.01	0.1274
sometimes	reference				
yes	**−0.09**	**0.91**	**0.86**	**0.98**	**0.0075**
**Appropriate amount of fluids for body weight**					
no	−0.005	1.00	0.93	1.07	0.8949
sometimes	reference				
yes	**−0.07**	**0.93**	**0.89**	**0.98**	**0.0096**
**Regular physical activity. at least 150 min a week**					
no	**0.08**	**1.09**	**1.03**	**1.16**	**0.0049**
sometimes	reference				
yes	**−0.15**	**0.86**	**0.81**	**0.91**	**<0.001**
**Sleep 6–8 h a day**					
no	**0.13**	**1.14**	**1.03**	**1.26**	**0.0098**
sometimes	reference				
yes	**−0.17**	**0.84**	**0.80**	**0.89**	**<0.001**
**Severely limiting alcohol consumption**					
no	−0.04	0.96	0.88	1.05	0.3582
sometimes	reference				
yes	**0.06**	**1.06**	**1.00**	**1.12**	**0.0407**
**Non smoking tobacco products**					
no	0.09	1.09	1.00	1.20	0.0597
sometimes	reference				
yes	**0.12**	**1.13**	**1.03**	**1.23**	**0.0067**
**Reducing/coping with stress**					
no	**0.14**	**1.15**	**1.07**	**1.23**	**<0.001**
sometimes	reference				
yes	**−0.21**	**0.81**	**0.77**	**0.85**	**<0.001**
**Using appropriate cosmetics/care**					
no	**−0.22**	**0.80**	**0.74**	**0.87**	**<0.001**
sometimes	reference				
yes	**−0.19**	**0.83**	**0.79**	**0.88**	**<0.001**
**Using treatments (massages. beauty treatments. aesthetic medicine treatments. etc.)**					
no	0.03	1.03	0.97	1.09	0.3622
sometimes	reference				
yes	**−0.26**	**0.77**	**0.71**	**0.84**	**<0.001**
**Avoiding fast food and sweetened (carbonated or not) drinks**					
no	0.01	1.01	0.93	1.11	0.7860
sometimes	reference				
yes	**−0.08**	**0.92**	**0.87**	**0.97**	**0.0045**
**I take dietary supplements**					
no	**−0.20**	**0.82**	**0.77**	**0.87**	**<0.001**
sometimes	reference				
yes	−0.005	1.00	0.94	1.05	0.8687
**Professional status**					
employed under an employment contract or running your own business	0.003	1.00	0.86	1.17	0.9667
employed under another contract (mandate contract. contract for specific work)	0.06	1.06	0.92	1.23	0.4235
running a farm	−0.11	0.89	0.71	1.12	0.3310
pensioner	reference				
unemployed with the right to benefits	−0.12	0.89	0.72	1.10	0.2834
unemployed without the right to benefits	**−0.13**	**0.88**	**0.78**	**0.99**	**0.0319**
not working/studying	**−0.16**	**0.85**	**0.78**	**0.93**	**<0.001**
**Body mass**	0.05	1.05	0.99	1.11	0.0940

**Table 5:** Results of logistic regression regarding sociodemographic and health-related factors associated with the presence of a burning sensation, as well as the odds ratio (OR) and its 95% confidence interval (CI) (in red: the most statistically significant (*p*-value < 0.05) predictors of the presence of burning sensation). The term ‘in red’ is bolded for emphasis.

Trait	Estimate	OR	Lower 95 CI OR	Uppper 95 CI OR	*p*
**Intercept 1**	**−1.73**	**0.18**	**0.15**	**0.22**	**<0.001**
**Intercept 2**	0.0001	1.00	0.82	1.21	0.9989
**Gender**					
men	**−0.26**	**0.77**	0.67	0.89	**<0.001**
women	reference				
**Age**					
18–24	−0.03	0.97	0.81	1.15	0.7029
25–34	−0.02	0.98	0.90	1.08	0.7285
35–44	0.03	1.03	0.95	1.11	0.4999
45–54	reference				
55–64	**−0.19**	**0.83**	**0.76**	**0.91**	**<0.001**
65+	**−0.23**	**0.80**	**0.70**	**0.90**	**<0.001**
**Education**					
primary and vocational	**−0.16**	**0.85**	**0.76**	**0.97**	**0.0118**
secondary and post-secondary	reference				
higher	0.04	1.04	0.98	1.11	0.1827
**Urbanization**					
countryside	−0.01	0.99	0.90	1.10	0.8954
city up to 19,000 citizens	0.05	1.05	0.94	1.17	0.3952
city of 20–49,000 citizens	0.04	1.04	0.94	1.15	0.4842
city of 50–99,000 citizens	reference				
city of 100–199,000 citizens	0.01	1.01	0.90	1.12	0.9151
city of 200–499,000 citizens	0.06	1.06	0.95	1.18	0.3073
city up to 500,000 or more citizens	**0.10**	**1.11**	**1.01**	**1.22**	**0.0390**
**A healthy, balanced diet**					
no	**−0.08**	**0.92**	**0.86**	**0.99**	**0.0306**
sometimes	reference				
yes	**−0.11**	**0.89**	**0.83**	**0.96**	**0.0011**
**At least five servings of vegetables and fruit**					
no	−0.04	0.96	0.90	1.02	0.1907
sometimes	reference				
yes	0.01	1.01	0.94	1.09	0.8430
**Appropriate amount of fluids for body weight**					
no	−0.02	0.98	0.91	1.06	0.6967
sometimes	reference				
yes	**−0.08**	**0.93**	**0.87**	**0.98**	**0.0099**
**Regular physical activity, at least 150 min a week**					
no	0.02	1.02	0.96	1.09	0.4758
sometimes	reference				
yes	**−0.16**	**0.85**	**0.80**	**0.91**	**<0.001**
**Sleep 6–8 h a day**					
no	**0.15**	**1.16**	**1.04**	**1.29**	**0.0057**
sometimes	reference				
yes	**−0.22**	**0.80**	**0.75**	**0.85**	**<0.001**
**Severely limiting alcohol consumption**					
no	−0.07	0.93	0.85	1.02	0.1400
sometimes	reference				
yes	0.04	1.04	0.98	1.11	0.1954
**Non smoking tobacco products**					
no	0.01	1.01	0.91	1.12	0.9149
sometimes	reference				
yes	−0.001	1.00	0.91	1.10	0.9884
**Reducing/coping with stress**					
no	**0.12**	**1.13**	**1.05**	**1.21**	**0.0014**
sometimes	reference				
yes	**−0.25**	**0.78**	**0.73**	**0.83**	**<0.001**
**Using appropriate cosmetics/care**					
no	**−0.25**	**0.78**	**0.71**	**0.86**	**<0.001**
sometimes	reference				
yes	−0.04	0.97	0.91	1.02	0.2450
**Using treatments (massages. beauty treatments. aesthetic medicine treatments. etc.)**					
no	−0.04	0.96	0.91	1.03	0.2568
sometimes	reference				
yes	**−0.22**	**0.80**	**0.73**	**0.88**	**<0.001**
**Avoiding fast food and sweetened (carbonated or not) drinks**					
no	−0.07	0.94	0.85	1.03	0.1897
sometimes	reference				
yes	**−0.10**	**0.90**	**0.85**	**0.96**	**0.0012**
**I take dietary supplements**					
no	**−0.19**	**0.83**	**0.78**	**0.88**	**<0.001**
sometimes	reference				
yes	0.04	1.04	0.98	1.11	0.2014
**Professional status**					
employed under an employment contract or running your own business	0.11	1.11	0.94	1.32	0.2196
employed under another contract (mandate contract. contract for specific work)	0.06	1.06	0.90	1.25	0.4897
running a farm	0.02	1.02	0.79	1.31	0.8697
pensioner	reference				
unemployed with the right to benefits	−0.04	0.96	0.75	1.21	0.7185
unemployed without the right to benefits	−0.05	0.95	0.83	1.09	0.4738
not working/studying	**−0.13**	**0.88**	**0.80**	**0.98**	**0.0168**
**Body mass**	0.06	1.06	1.00	1.13	0.0692

**Table 6:** Results of logistic regression regarding sociodemographic and health-related factors associated with the presence of rash, as well as the odds ratio (OR) and its 95% confidence interval (CI) (in red: the most statistically significant (*p*-value < 0.05) predictors of the presence of skin redness). The term ‘in red’ is bolded for emphasis.

Trait	Estimate	OR	Lower 95 CI OR	Uppper 95 CI OR	*p*
**Intercept 1**	**−1.02**	**0.36**	**0.30**	**0.43**	**<0.001**
**Intercept 2**	**0.71**	**2.04**	**1.71**	**2.43**	**<0.001**
**Gender**					
men	**−0.30**	**0.74**	**0.65**	**0.84**	**<0.001**
women	reference				
**Age (years)**					
18–24	**0.47**	**1.60**	**1.36**	**1.87**	**<0.001**
25–34	**0.31**	**1.36**	**1.25**	**1.48**	**<0.001**
35–44	**0.17**	**1.19**	**1.11**	**1.27**	**<0.001**
45–54	reference				
55–64	**−0.24**	**0.79**	**0.72**	**0.85**	**<0.001**
65+	**−0.39**	**0.68**	**0.60**	**0.76**	**<0.001**
**Education**					
primary and vocational	**−0.33**	**0.72**	**0.64**	**0.81**	**<0.001**
secondary and post-secondary	reference				
higher	**0.16**	**1.17**	**1.10**	**1.24**	**<0.001**
**Urbanization**					
countryside	−0.08	0.92	0.84	1.01	0.0876
city up to 19,000 citizens	−0.02	0.98	0.89	1.08	0.7329
city of 20–49,000 citizens	0.03	1.03	0.94	1.13	0.4865
city of 50–99,000 citizens	reference				
city of 100–199,000 citizens	**0.10**	**1.11**	**1.00**	**1.22**	**0.0449**
city of 200–499,000 citizens	**0.16**	**1.17**	**1.06**	**1.29**	**0.0016**
city of 500,000 or more citizens	**0.16**	**1.18**	**1.08**	**1.29**	**<0.001**
**A healthy, balanced diet**					
no	**−0.14**	**0.87**	**0.81**	**0.93**	**<0.001**
sometimes	reference				
yes	**−0.11**	**0.90**	**0.84**	**0.95**	**<0.001**
**At least five servings of vegetables and fruit**					
no	−0.03	0.97	0.91	1.03	0.2840
sometimes	reference				
yes	−0.02	0.98	0.92	1.05	0.6553
**Appropriate amount of fluids for body weight**					
no	0.05	1.05	0.97	1.13	0.2156
sometimes	reference				
yes	**−0.08**	**0.92**	**0.87**	**0.97**	**0.0017**
**Regular physical activity. at least 150 min a week**					
no	0.02	1.02	0.96	1.09	0.4457
sometimes	reference				
yes	**−0.11**	**0.90**	**0.85**	**0.95**	**<0.001**
**Sleep 6–8 h a day**					
no	0.07	1.07	0.97	1.18	0.1781
sometimes	reference				
yes	**−0.19**	**0.83**	**0.78**	**0.87**	**<0.001**
**Severely limiting alcohol consumption**					
no	−0.02	0.98	0.90	1.07	0.7138
sometimes	reference				
yes	−0.05	0.95	0.90	1.01	0.1079
**Nonsmoking tobacco products**					
no	−0.03	0.98	0.89	1.07	0.5976
sometimes	reference				
yes	0.06	1.06	0.97	1.16	0.2113
**Reducing/coping with stress**					
no	**0.16**	**1.17**	**1.10**	**1.26**	**<0.001**
sometimes	reference				
yes	**−0.25**	**0.78**	**0.74**	**0.82**	**<0.001**
**Using appropriate cosmetics/care**					
no	**−0.42**	**0.66**	**0.61**	**0.72**	**<0.001**
sometimes	reference				
yes	0.03	1.03	0.98	1.09	0.2346
**Using treatments (massages. beauty treatments. aesthetic medicine treatments. etc.)**					
no	**−0.06**	**0.94**	**0.89**	**0.99**	**0.0250**
sometimes	reference				
yes	**−0.16**	**0.86**	**0.79**	**0.93**	**<0.001**
**Avoiding fast food and sweetened (carbonated or not) drinks**					
no	−0.03	0.97	0.89	1.06	0.5432
sometimes	reference				
yes	**−0.11**	**0.90**	**0.84**	**0.95**	**<0.001**
**I take dietary supplements**					
no	**−0.19**	**0.83**	**0.78**	**0.88**	**<0.001**
sometimes	reference				
yes	0.04	1.04	0.98	1.10	0.1891
**Professional status**					
employed under an employment contract or running your own business	**0.26**	**1.30**	**1.11**	**1.52**	**0.0010**
employed under another contract (mandate contract. contract for specific work)	**0.22**	**1.25**	**1.07**	**1.45**	**0.0038**
running a farm	−0.04	0.96	0.76	1.21	0.7354
pensioner	reference				
unemployed with the right to benefits	0.07	1.07	0.86	1.33	0.5226
unemployed without the right to benefits	0.08	1.09	0.96	1.23	0.1774
not working/studying	0.04	1.04	0.95	1.14	0.4006
**Body mass**	**0.07**	**1.07**	**1.01**	**1.13**	**0.0212**

**Table 7:** Results of logistic regression regarding sociodemographic and health-related factors associated with the presence of burning sensation, as well as the odds ratio (OR) and its 95% confidence interval (CI) (in red: the most statistically significant (*p*-value < 0.05) predictors of the presence of burning sensation). The term ‘in red’ is bolded for emphasis.

Trait	Estimate	OR	Lower 95 CI OR	Uppper 95 CI OR	*p*
**Intercept 1**	**−1.73**	**0.18**	**0.15**	**0.22**	**<0.001**
**Intercept 2**	0.0001	1.00	0.82	1.21	0.9989
**Gender**					
men	**−0.26**	**0.77**	0.67	0.89	**<0.001**
women	reference				
**Age**					
18–24	−0.03	0.97	0.81	1.15	0.7029
25–34	−0.02	0.98	0.90	1.08	0.7285
35–44	0.03	1.03	0.95	1.11	0.4999
45–54	reference				
55–64	**−0.19**	**0.83**	**0.76**	**0.91**	**<0.001**
65+	**−0.23**	**0.80**	**0.70**	**0.90**	**<0.001**
**Education**					
primary and vocational	**−0.16**	**0.85**	**0.76**	**0.97**	**0.0118**
secondary and post-secondary	reference				
higher	0.04	1.04	0.98	1.11	0.1827
**Urbanization**					
countryside	−0.01	0.99	0.90	1.10	0.8954
city up to 19,000 citizens	0.05	1.05	0.94	1.17	0.3952
city of 20–49,000 citizens	0.04	1.04	0.94	1.15	0.4842
city of 50–99,000 citizens	reference				
city of 100–199,000 citizens	0.01	1.01	0.90	1.12	0.9151
city of 200–499,000 citizens	0.06	1.06	0.95	1.18	0.3073
city up to 500,000 or more citizens	**0.10**	**1.11**	**1.01**	**1.22**	**0.0390**
**A healthy, balanced diet**					
no	**−0.08**	**0.92**	**0.86**	**0.99**	**0.0306**
sometimes	reference				
yes	**−0.11**	**0.89**	**0.83**	**0.96**	**0.0011**
**At least five servings of vegetables and fruit**					
no	−0.04	0.96	0.90	1.02	0.1907
sometimes	reference				
yes	0.01	1.01	0.94	1.09	0.8430
**Appropriate amount of fluids for body weight**					
no	−0.02	0.98	0.91	1.06	0.6967
sometimes	reference				
yes	**−0.08**	**0.93**	**0.87**	**0.98**	**0.0099**
**Regular physical activity, at least 150 min a week**					
no	0.02	1.02	0.96	1.09	0.4758
sometimes	reference				
yes	**−0.16**	**0.85**	**0.80**	**0.91**	**<0.001**
**Sleep 6–8 h a day**					
no	**0.15**	**1.16**	**1.04**	**1.29**	**0.0057**
sometimes	reference				
yes	**−0.22**	**0.80**	**0.75**	**0.85**	**<0.001**
**Severely limiting alcohol consumption**					
no	−0.07	0.93	0.85	1.02	0.1400
sometimes	reference				
yes	0.04	1.04	0.98	1.11	0.1954
**Non smoking tobacco products**					
no	0.01	1.01	0.91	1.12	0.9149
sometimes	reference				
yes	−0.001	1.00	0.91	1.10	0.9884
**Reducing/coping with stress**					
no	**0.12**	**1.13**	**1.05**	**1.21**	**0.0014**
sometimes	reference				
yes	**−0.25**	**0.78**	**0.73**	**0.83**	**<0.001**
**Using appropriate cosmetics/care**					
no	**−0.25**	**0.78**	**0.71**	**0.86**	**<0.001**
sometimes	reference				
yes	−0.04	0.97	0.91	1.02	0.2450
**Using treatments (massages. beauty treatments. aesthetic medicine treatments. etc.)**					
no	−0.04	0.96	0.91	1.03	0.2568
sometimes	reference				
yes	**−0.22**	**0.80**	**0.73**	**0.88**	**<0.001**
**Avoiding fast food and sweetened (carbonated or not) drinks**					
no	−0.07	0.94	0.85	1.03	0.1897
sometimes	reference				
yes	**−0.10**	**0.90**	**0.85**	**0.96**	**0.0012**
**I take dietary supplements**					
no	**−0.19**	**0.83**	**0.78**	**0.88**	**<0.001**
sometimes	reference				
yes	0.04	1.04	0.98	1.11	0.2014
**Professional status**					
employed under an employment contract or running your own business	0.11	1.11	0.94	1.32	0.2196
employed under another contract (mandate contract. contract for specific work)	0.06	1.06	0.90	1.25	0.4897
running a farm	0.02	1.02	0.79	1.31	0.8697
pensioner	reference				
unemployed with the right to benefits	−0.04	0.96	0.75	1.21	0.7185
unemployed without the right to benefits	−0.05	0.95	0.83	1.09	0.4738
not working/studying	**−0.13**	**0.88**	**0.80**	**0.98**	**0.0168**
**Body mass**	0.06	1.06	1.00	1.13	0.0692

**Table 8:** Results of logistic regression regarding sociodemographic and health-related factors associated with desquamation, as well as the odds ratio (OR) and its 95% confidence interval (CI) (in red: the most statistically significant (*p*-value < 0.05) predictors of the presence of peeling). The term ‘in red’ is bolded for emphasis.

Trait	Estimate	OR	Lower 95 CI OR	Uppper 95 CI OR	*p*
**Intercept 1**	**−1.51**	**0.22**	**0.18**	**0.27**	**<0.001**
**Intercept 2**	0.01	1.01	0.83	1.22	0.9184
**Gender**					
men	−0.04	0.96	0.84	1.10	0.5303
women	reference				
**Age**					
18–24	**0.28**	**1.32**	**1.11**	**1.57**	**0.0016**
25–34	**0.30**	**1.35**	**1.24**	**1.48**	**<0.001**
35–44	**0.19**	**1.21**	**1.12**	**1.30**	**<0.001**
45–54	reference				
55–64	**−0.13**	**0.88**	**0.80**	**0.96**	**0.0057**
65+	**−0.22**	**0.80**	**0.71**	**0.91**	**<0.001**
**Education**					
primary and vocational	**−0.20**	**0.82**	**0.72**	**0.93**	**0.0015**
secondary and post-secondary	reference				
higher	**0.22**	**1.24**	**1.16**	**1.32**	**<0.001**
**Urbanization**					
countryside	**−0.16**	**0.85**	**0.77**	**0.94**	**0.0021**
city up to 19,000 citizens	−0.07	0.93	0.84	1.03	0.1634
city of 20–49,000 citizens	−0.05	0.95	0.86	1.05	0.3215
city of 50–99,000 citizens	reference				
city of 100–199,000 citizens	−0.003	1.00	0.90	1.11	0.9507
city of 200–499,000 citizens	−0.05	0.95	0.86	1.05	0.3408
city of 500,000 or more citizens	−0.04	0.96	0.88	1.06	0.4252
**A healthy, balanced diet**					
no	**−0.10**	**0.91**	**0.85**	**0.98**	**0.0090**
sometimes	reference				
yes	**−0.08**	**0.92**	**0.86**	**0.99**	**0.0225**
**At least five servings of vegetables and fruit**					
no	−0.002	1.00	0.94	1.06	0.9591
sometimes	reference				
yes	−0.05	0.95	0.88	1.02	0.1723
**Appropriate amount of fluids for body weight**					
no	0.02	1.02	0.94	1.10	0.6878
sometimes	reference				
yes	**−0.07**	**0.93**	**0.88**	**0.98**	**0.0108**
**Regular physical activity. at least 150 min a week**					
no	0.03	1.03	0.96	1.10	0.4140
sometimes	reference				
yes	**−0.17**	**0.84**	**0.79**	**0.89**	**<0.001**
**Sleep 6–8 h a day**					
no	−0.02	0.98	0.88	1.08	0.6448
sometimes	reference				
yes	**−0.19**	**0.82**	**0.78**	**0.87**	**<0.001**
**Severely limiting alcohol consumption**					
no	−0.03	0.97	0.89	1.07	0.5698
sometimes	reference				
yes	**0.07**	**1.07**	**1.01**	**1.14**	**0.0326**
**Non smoking tobacco products**					
no	−0.06	0.94	0.85	1.04	0.2181
sometimes	reference				
yes	−0.01	0.99	0.90	1.09	0.7705
**Reducing/coping with stress**					
no	**0.14**	**1.15**	**1.07**	**1.23**	**<0.001**
sometimes	reference				
yes	**−0.18**	**0.84**	**0.79**	**0.89**	**<0.001**
**Using appropriate cosmetics/care**					
no	**−0.29**	**0.75**	**0.68**	**0.82**	**<0.001**
sometimes	reference				
yes	**−0.19**	**0.83**	**0.78**	**0.88**	**<0.001**
**Using treatments (massages. beauty treatments. aesthetic medicine treatments. etc.)**					
no	0.004	1.00	0.94	1.07	0.8948
sometimes	reference				
yes	**−0.17**	**0.84**	**0.77**	**0.93**	**<0.001**
**Avoiding fast food and sweetened (carbonated or not) drinks**					
no	−0.03	0.97	0.88	1.06	0.4870
sometimes	reference				
yes	0.01	1.01	0.95	1.07	0.7561
**I take dietary supplements**					
no	**−0.23**	**0.79**	**0.74**	**0.84**	**<0.001**
sometimes	reference				
yes	−0.02	0.98	0.92	1.04	0.4299
**Professional status**					
employed under an employment contract or running your own business	−0.05	0.95	0.80	1.12	0.5295
employed under another contract (mandate contract. contract for specific work)	−0.0002	1.00	0.85	1.18	0.9983
running a farm	−0.22	0.81	0.62	1.04	0.0955
pensioner	reference				
unemployed with the right to benefits	**−0.33**	**0.72**	**0.56**	**0.92**	**0.0095**
unemployed without the right to benefits	**−0.17**	**0.84**	**0.74**	**0.97**	**0.0143**
not working/studying	**−0.15**	**0.86**	**0.78**	**0.95**	**0.0042**
**Body mass**	0.02	1.02	0.96	1.09	0.4507

**Table 9:** Results of logistic regression regarding sociodemographic and health-related factors associated with the presence of sunburn, as well as the odds ratio (OR) and its 95% confidence interval (CI) (in red: the most statistically significant (*p*-value < 0.05) predictors of the presence of sunburn). The term ‘in red’ is bolded for emphasis.

Trait	Estimate	OR	Lower 95 CI OR	Uppper 95 CI OR	*p*
**Intercept 1**	**−3.46**	**0.03**	**0.03**	**0.04**	**<0.001**
**Intercept 2**	0.06	1.06	0.87	1.29	0.5477
**Gender**					
men	**−0.36**	**0.70**	**0.61**	**0.80**	**<0.001**
women	reference				
**Age**					
18–24	**−0.33**	**0.72**	**0.60**	**0.86**	**<0.001**
25–34	**−0.14**	**0.87**	**0.79**	**0.95**	**0.0021**
35–44	0.04	1.04	0.97	1.12	0.2700
45–54	reference				
55–64	−0.08	0.92	0.84	1.01	0.0904
65+	**−0.28**	**0.75**	**0.67**	**0.85**	**<0.001**
**Education**					
primary and vocational	**−0.26**	**0.77**	**0.68**	**0.87**	**<0.001**
secondary and post-secondary	reference				
higher	**0.22**	**1.25**	**1.17**	**1.34**	**<0.001**
**Urbanization**					
countryside	−0.04	0.96	0.87	1.06	0.4379
city up to 19,000 citizens	−0.06	0.94	0.84	1.05	0.2526
city of 20–49,000 citizens	0.005	1.00	0.91	1.11	0.9251
city of 50–99,000 citizens	reference				
city of 100–199,000 citizens	−0.01	0.99	0.89	1.11	0.8577
city of 200–499,000 citizens	0.03	1.03	0.92	1.14	0.6225
city up to 500,000 or more citizens	**0.16**	**1.17**	**1.06**	**1.29**	**0.0016**
**A healthy, balanced diet**					
no	**−0.19**	**0.83**	**0.77**	**0.89**	**<0.001**
sometimes	reference				
yes	**−0.09**	**0.91**	**0.85**	**0.98**	**0.0075**
**At least five servings of vegetables and fruit**					
no	−0.05	0.95	0.89	1.01	0.1210
sometimes	reference				
yes	−0.06	0.94	0.87	1.01	0.1122
**Appropriate amount of fluids for body weight**					
no	0.02	1.02	0.94	1.10	0.6224
sometimes	reference				
yes	**−0.12**	**0.88**	**0.83**	**0.94**	**<0.001**
**Regular physical activity, at least 150 min a week**					
no	**−0.14**	**0.87**	**0.81**	**0.93**	**<0.001**
sometimes	reference				
yes	**−0.07**	**0.93**	**0.87**	**0.99**	**0.0218**
**Sleep 6–8 h a day**					
no	−0.01	0.99	0.89	1.10	0.8315
sometimes	reference				
yes	**−0.13**	**0.88**	**0.83**	**0.93**	**<0.001**
**Severely limiting alcohol consumption**					
no	−0.05	0.95	0.86	1.04	0.2638
sometimes	reference				
yes	**−0.17**	**0.84**	**0.79**	**0.90**	**<0.001**
**Non smoking tobacco products**					
no	0.04	1.04	0.93	1.15	0.5062
sometimes	reference				
yes	**0.17**	**1.18**	**1.07**	**1.31**	**<0.001**
**Reducing/coping with stress**					
no	**0.09**	**1.09**	**1.02**	**1.18**	**0.0168**
sometimes	reference				
yes	**−0.18**	**0.84**	**0.79**	**0.89**	**<0.001**
**Using appropriate cosmetics/care**					
no	**−0.18**	**0.84**	**0.76**	**0.92**	**<0.001**
sometimes	reference				
yes	**−0.09**	**0.92**	**0.86**	**0.97**	**0.0041**
**Using treatments (massages, beauty treatments, aesthetic medicine treatments, etc.)**					
no	**−0.16**	**0.85**	**0.80**	**0.91**	**<0.001**
sometimes	reference				
yes	**−0.23**	**0.80**	**0.73**	**0.87**	**<0.001**
**Avoiding fast food and sweetened (carbonated or not) drinks**					
no	0.02	1.02	0.92	1.12	0.7162
sometimes	reference				
yes	**−0.14**	**0.87**	**0.82**	**0.93**	**<0.001**
**I take dietary supplements**					
no	**−0.28**	**0.75**	**0.71**	**0.80**	**<0.001**
sometimes	reference				
yes	**−0.11**	**0.89**	**0.84**	**0.95**	**0.0004**
**Professional status**					
employed under an employment contract or running your own business	**0.31**	**1.37**	**1.15**	**1.63**	**<0.001**
employed under another contract (mandate contract. contract for specific work)	**0.18**	**1.20**	**1.02**	**1.42**	**0.0301**
running a farm	0.14	1.15	0.89	1.49	0.2845
pensioner	reference				
unemployed with the right to benefits	**0.25**	**1.29**	**1.02**	**1.63**	**0.0368**
unemployed without the right to benefits	0.11	1.11	0.97	1.28	0.1252
not working/studying	**0.12**	**1.12**	**1.01**	**1.25**	**0.0276**
**Body mass**	**0.12**	**1.12**	**1.06**	**1.20**	**<0.001**

## Data Availability

The original contributions presented in this study are included in the article. Further inquiries can be directed to the corresponding authors.
